# Anticancer therapeutic strategies for targeting mutant p53-Y220C

**DOI:** 10.7555/JBR.37.20230093

**Published:** 2024-05-13

**Authors:** Vitaly Chasov, Damir Davletshin, Elvina Gilyazova, Regina Mirgayazova, Anna Kudriaeva, Raniya Khadiullina, Youyong Yuan, Emil Bulatov

**Affiliations:** 1 Institute of Fundamental Medicine and Biology, Kazan Federal University, Kazan 420008, Russia; 2 Shemyakin-Ovchinnikov Institute of Bioorganic Chemistry, Russian Academy of Sciences, Moscow 117997, Russia; 3 Institute of Life Sciences, School of Medicine, South China University of Technology, Guangzhou, Guangdong 510006, China

**Keywords:** p53, Y220C mutation, small molecule, DNA-binding domain, immunotherapy, T cell receptor mimic antibody

## Abstract

The tumor suppressor p53 is a transcription factor with a powerful antitumor activity that is controlled by its negative regulator murine double minute 2 (MDM2, also termed HDM2 in humans) through a feedback mechanism. At the same time, *TP53* is the most frequently mutated gene in human cancers. Mutant p53 proteins lose wild-type p53 tumor suppression functions but acquire new oncogenic properties, among which are deregulating cell proliferation, increasing chemoresistance, disrupting tissue architecture, and promoting migration, invasion and metastasis as well as several other pro-oncogenic activities. The oncogenic p53 mutation Y220C creates an extended surface crevice in the DNA-binding domain destabilizing p53 and causing its denaturation and aggregation. This cavity accommodates stabilizing small molecules that have therapeutic values. The development of suitable small-molecule stabilizers is one of the therapeutic strategies for reactivating the Y220C mutant protein. In this review, we summarize approaches that target p53-Y220C, including reactivating this mutation with small molecules that bind Y220C to the hydrophobic pocket and developing immunotherapies as the goal for the near future, which target tumor cells that express the p53-Y220C neoantigen.

## Introduction

The tumor suppressor protein p53 is a tetrameric nuclear protein that executes its functions through transcriptional regulation of genes involved in DNA repair, senescence, and apoptosis^[1–2]^. p53 is known to be the "guardian of the genome" that prevents the proliferation of cells harboring genetic aberrations, and plays an important role in preventing the division of cells carrying the mutated genes^[3]^. In response to stress, hypoxia, or DNA damage, p53 translocates from the cytoplasm to the nucleus, where it activates many genes required for DNA repair. If DNA damage is severe, p53 induces the expression of apoptotic proteins^[2]^. The critical event leading to the activation of p53 is the phosphorylation of its N-terminal domain^[4]^. The N-terminal transcriptional activation domain contains a large number of phosphorylation sites and is considered the primary target for protein kinases transducing stress signals^[5]^. The p53 level in cells is controlled by murine double minute 2 (MDM2) at different levels: 1) *via* ubiquitination followed by proteasomal degradation (ubiquitin-proteasome machinery); 2) inhibition of transcriptional activation of p53 through the induction of its export to the cytoplasm; and 3) the attenuation of p53 binding to its target DNA sequences^[6]^. The activation of p53 leads to the overexpression of its negative regulator, MDM2; therefore, it is considered an autoregulatory loop^[7]^.

The *TP53* gene, encoding the p53 protein, is the most frequently altered gene in human tumors^[8]^. The loss of wild-type (WT) p53 functions is the primary outcome of *TP53* mutations to deprive cells of intrinsic tumor suppressive responses, such as senescence and apoptosis^[9]^. The majority of *TP53* mutations are missense, producing single residue substitutions within the protein's DNA-binding domains (DBDs), and are classified as either contact or conformational mutations^[10]^. p53 missense mutant proteins lose the ability to activate canonical p53 target genes, and some mutants exert *trans*-dominant repression over the WT counterpart. Beyond this, cancer cells appear to gain selective advantages by retaining only the mutant form of the p53 protein. Specific missense p53 mutants have been reported to subvert crucial cellular pathways, foster cancer cell proliferation and survival, and promote invasion, migration, metastasis, and chemoresistance^[10]^.

p53 is inactivated by mutations in approximately 50% of all human cancers, with the majority of point mutations occurring in its DBDs^[11]^. These mutations affect DNA binding and/or thermodynamic stability of p53^[10]^. Approximately one-third of these mutants are simply unstable and undergo rapid denaturation under physiological conditions^[12]^. Importantly, many of these destabilized p53 mutants display transcriptional activities at sub-physiological temperatures^[13–14]^, suggesting that their functions may be restored by the binding small molecules that stabilize the structure^[15]^.

Y220C is one of the most frequently occurring cancer mutations^[16]^. The mutation site is far away from the functional region of p53 and does not critically change the overall protein structure^[15]^. The Y220C mutant provides a particularly suitable case for the development of small-molecule stabilizers. Replacement of tyrosine by cysteine in this mutation creates a narrow, hydrophobic pocket on the surface of the p53 DBD, which reduces its thermal stability by approximately 4 kcal/mol^[17]^. While the WT p53 is moderately stable, melting at 44 ℃^[12]^, the Y220C mutant rapidly unfolds under physiological conditions, which effectively abrogates p53 signaling and drives tumorigenesis^[18]^. Importantly, the mutation-induced crevice is distant from the p53 surfaces involved in DNA recognition or protein-protein interactions, allowing for the development of stabilizing small molecules without interfering with the binding to its natural substrates. Recent studies suggest that the p53-Y220C mutation is immunogenic and induces intratumoral T cell responses to specific neoantigens, making it an attractive target for immunotherapies^[19–21]^. In the present review, we summarize current strategies for the reactivation of mutant p53-Y220C as well as a potential novel anticancer immunotherapy approach.

## p53 mutations

**Figure 1:**
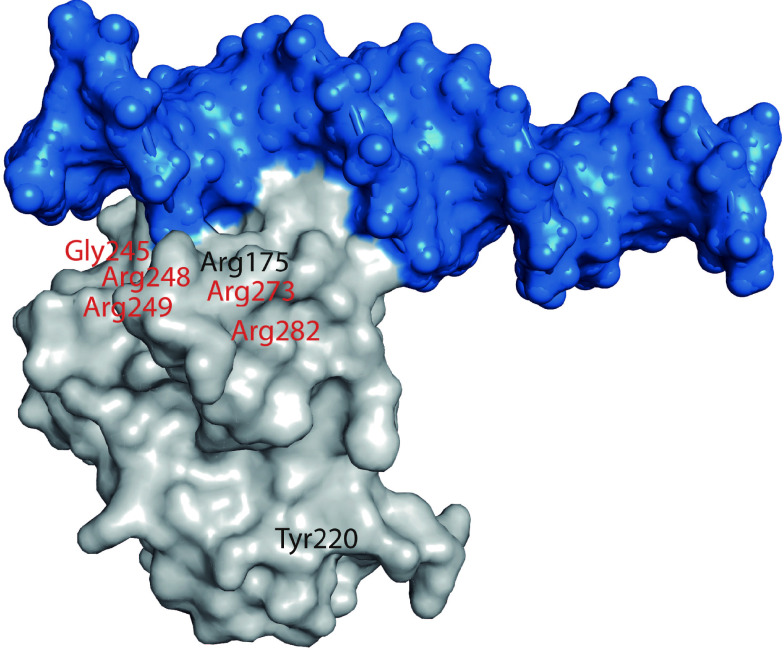
Crystal structure of p53 core domain (gray) in complex with DNA (blue) (PDB code 4HJE).

While many other tumor suppressor genes are predominantly inactivated through the deletion or truncating mutations in cancer, the majority of p53 mutations in human cancers are missense mutations that lead to the expression of full-length mutant p53 proteins with the substitution of a single amino acid. Certain "hotspot" residues are mutated at a higher frequency. Six "hotspot" mutations, *i.e.*, R175, G245, R248, R249, R273, and R282, represent about 30% of all mutations in *TP53* across all human cancer types (***Fig. 1***)^[22–24]^.

DNA contact mutations occur in the amino acid residues that make a direct contact with p53 target DNA sequences and are critical for DNA binding, whereas conformational mutations alter the structure of p53 to abolish its DNA-binding ability. DNA-contact mutations impair p53-DNA binding by eliminating essential protein-DNA contacts, such as a mutation of Arg248 or Arg273^[22,25]^. Conformational mutations destabilize the DBD that has a relatively low intrinsic kinetic and thermodynamic stability, so that it rapidly unfolds at physiological temperature, followed by aggregation^[13,17,26]^.

Both contact and conformational mutants not only lose transcriptional activities but also exhibit dominant-negative effects on the remaining WT p53 allele, in addition to the homologous tumor suppressors p63 and p73^[27]^. Mutant p53 proteins form a tetramer with the WT p53, which blocks the function of the remaining WT p53 in tumor suppression^[9,28]^.

There are two types of mutations that interfere with gene functions. The loss-of-function mutation is a type of mutation in which the altered gene product lacks molecular functions of the WT entity, while the gain-of-function mutation is a type of mutation in which the altered gene product possesses a new molecular function or a new pattern of gene expression. The loss of WT p53 functions is the primary outcome of *TP53* mutations, while the gain-of-function mutant p53 proteins enhance tumor progression, metastatic potential, and drug resistance, greatly contributing to malignant properties of cancer cells^[29]^. Although most p53 mutants lose their transcriptional activities and tumor-suppressive functions, some evidence suggests that sequence-specific p53 transcriptional activities may be restored in the mutant p53. For example, many p53 mutants are temperature-sensitive and restore their activities at permissive temperatures^[30]^. Furthermore, the p53 transcriptional activities have been restored by the insertion of second-site mutations or N-terminal deletion in several p53 mutants^[18]^. These data indicate the possibility of discovering natural or synthetic substances (peptides, small molecules, *etc*.) that may stabilize the mutant p53 in its active biological conformation, thus restoring its DNA-binding ability and rescuing the WT p53 activity.

The C-terminus of p53 contains a single Zn^2+^ ion that is required for proper protein folding and functions^[31]^. Mutations in this domain disrupt the protein stability and cause a loss of Zn^2+[32]^. Consequently, the discovery of small molecule Zn^2+^ chaperones, aimed at restoring the WT function in the mutant p53, has generated a significant attention. For example, the thiosemicarbazone family compound NSC319726 (ZMC1) has been shown to promote a "WT-like" conformational change in the p53R175H mutation, thereby restoring p53 transactivation function^[33]^. ZMC-1 buffers the intracellular free zinc levels to promote zinc binding, and therefore facilitates the proper folding of the mutant p53^[34]^. Other studies indicate that subtle tuning of the Zn-binding affinity of the metallochaperones is critical for p53 reactivation, and that, more broadly, the targeted metal ion chelation and redistribution have been shown as a promising anti-cancer strategy^[35]^.

Mutations in the p53 DBD result in structural destabilization, such as the protein unfolding at or below physiological temperatures, including the frequently occurring Y220C mutant^[36]^. The WT-specific conformational antibody Pab1620 was revealed to immunoprecipitate p53-Y220C at 28 ℃ but not 37 ℃, confirming the unfolding of the p53 DBD at the physiological temperature and a proper folding at the permissive temperature^[37]^. In addition to protein unfolding, p53-Y220C is prone to the loss of Zn^2+^ in the DBD, presenting an opportunity for drug design^[38–39]^. Outside the DNA-binding surface of p53, Y220C is the most common cancerogenic mutation. The Y220C mutation creates an extended cavity on the surface of the protein that destabilizes p53, causing denaturation and aggregation. This pocket is away from the surface involved in DNA binding and has a hydrophobic nature, making it an attractive site for targeting by small molecules^[15]^.

Molecular dynamics simulations, with Markov state models and nuclear magnetic resonance studies of the WT p53 and the Y220C mutant, have uncovered the involvement of the loop L6 in the slowest motions of the protein^[40]^. The loop L6 (residues 221–230, also termed the S7/S8 loop) is adjacent to the Y220C mutation, and the mutation-induced stabilization of alternate L6 conformations is distinct from all experimentally determined structures, in which the loop is both extended and located further away from the DNA-interacting surface. Finally, the simulations reveal a novel Y220C cryptic pocket nestled in the extended conformation of L6, which may be targeted for p53 rescue efforts^[40]^. Subsequently, the existence of this novel pocket was experimentally confirmed. Co-crystal structures of p53-Y220C with the small molecule activators of the methacrylamide indole series have been obtained, revealing two conformational states of the loop L6^[37]^. This information is very important for the improvement of approaches to the development of new mutant-specific drugs.

## Small molecule reactivators

**Figure 2:**
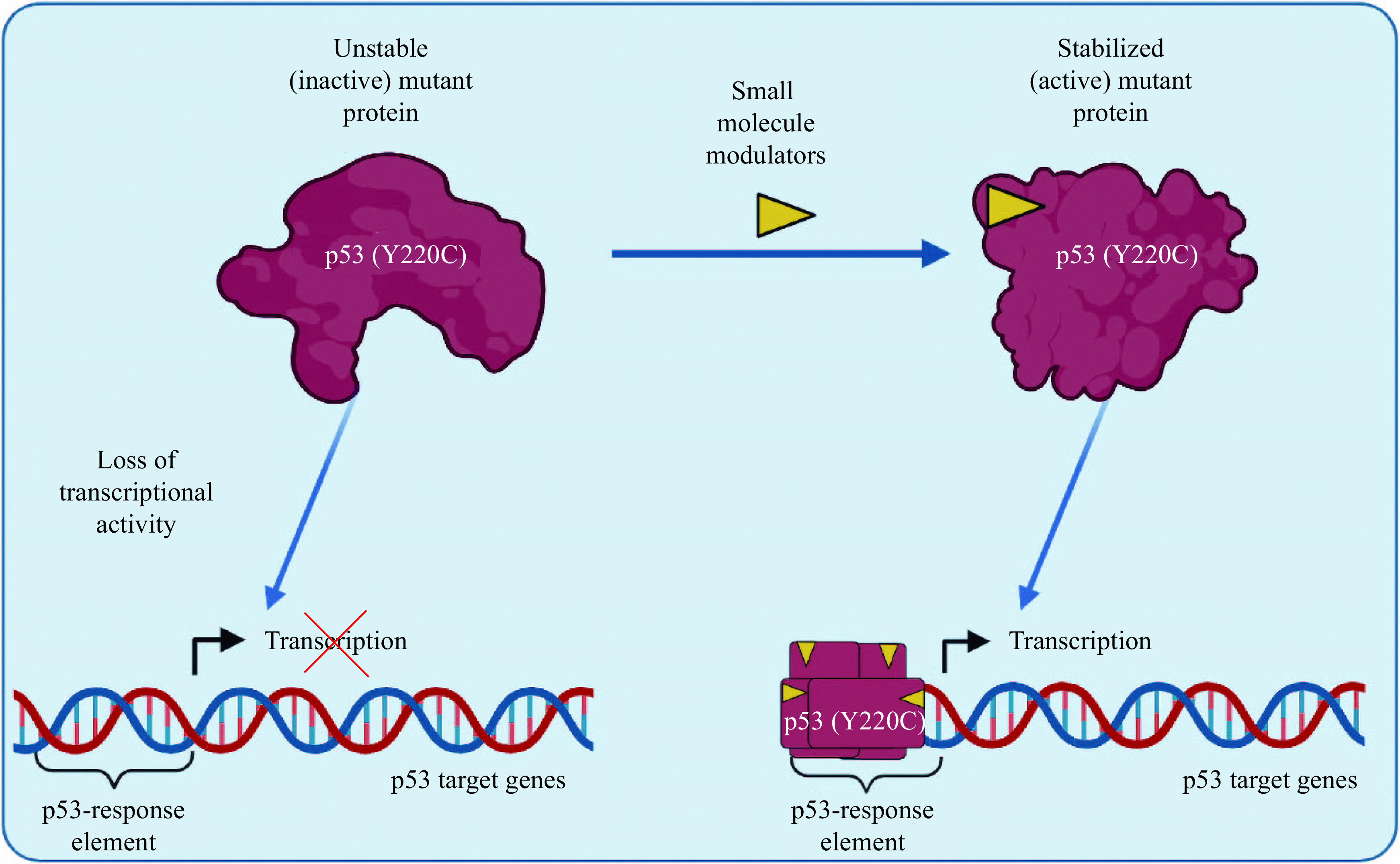
A therapeutic strategy to target p53-Y220C mutant by reactivation using small molecules.

In approximately 50% of human cancers, p53 retains its WT status, but its negative regulator, MDM2, is overexpressed, followed by deregulation of p53 levels. Blocking the p53-MDM2 interaction using small molecules is one of the therapeutic strategies, which has been demonstrated a significant progress in the past years^[41–42]^. However, MDM2 inhibitors were found to be ineffective in inducing activation of the mutated p53^[43]^. *Via* the Y220C mutation, Nutlin-3a, a potent inhibitor of the p53-MDM2 interaction, was observed to be unable to stimulate the p53-Y220C activity, even under the condition of therapeutic hypothermia, when the mutant was folded in a WT-like state^[44]^. Therefore, direct targeting of the p53 protein is a very attractive anticancer treatment approach. While all p53 mutations are potentially druggable, this task is complicated by the existence of over 2000 possible mutant p53 variants. Since contact mutations contain residues that interact directly with DNA, a successful targeting requires the insertion of interactions to compensate for missing DNA contacts^[36]^. Because of the lack of well-defined pockets in these contact p53 mutants, targeting them with small molecules is very difficult^[22]^. However, structural mutants contain mutations in residues that affect the thermal stability and structure of p53, rendering the mutants unstable at physiological temperatures and preventing them from achieving normal folding and WT-like activities^[10]^. From this perspective, small molecules that directly stabilize and restore the levels of correctly folded p53 may offer a more practical approach for targeting structural p53 mutants. This approach has been pursued, and one of the most attractive conformational mutations is Y220C, located in the C-terminus of p53, which is found in approximately 100000 new cases of cancer each year^[15]^ (***Fig. 2***).

One example of small molecules is APR-246 that has shown some positive results in recent phase Ⅰ/Ⅱ clinical trials^[45]^. APR-246 is a more potent methylated derivative of p53 reactivation and massive apoptosis-1 (PRIMA-1) induction, and is discovered by screening for compounds that both inhibit a mutant-p53-dependent cell growth and reactivate the mutant p53^[46–47]^. APR-246 is a pro-drug and works by its conversion in cells into the reactive electrophile methylene quinuclidinone (MQ) that binds covalently to cysteine residues of the mutant p53, resulting in protein reactivation^[48]^. While the exact mechanism of p53 reactivation by APR-246/MQ is not fully understood, it has been reported that MQ reacts with cysteine residues C124, C229, and C277 in the core of mutant p53 proteins, thereby changing their conformation from mutant to WT and resulting in the restoration of transactivation of WT p53 target genes that inhibit tumor growth^[49–50]^. Because of the non-specific nature of this process, MQ has been shown to affect p53 proteins in both conformational and contact mutants, and also bind to other proteins in cells, including many enzymes, and the modification of thioredoxin reductase 1 (TrxR1) leads to an increased production of reactive oxygen species, an additional cytotoxic mechanism^[51]^. The formation of amyloid aggregates because of mutations in p53 is known to promote tumor progression^[52]^. An unprecedented inhibition of the aggregation of Y220C mutant p53 by PRIMA-1 in hepatocellular carcinoma cell lines was observed, and this effect was particularly evident when PRIMA-1 was used in combination with cisplatin^[53]^. It has been reported that APR-246 eliminates cancer cells independently of p53 mutations by inducing different cell death pathways, including apoptosis, necroptosis, and ferroptosis, demonstrating that clinical testing of APR-246 should be broadened to include malignancies expressing WT p53, rather than being limited to cancers expressing the mutant p53^[54]^.

COTI-2 is a third-generation thiosemicarbazone compound discovered using a proprietary computational platform known as CHEMSAS^®^ that uses a unique combination of traditional and modern pharmacology principles, statistical modeling, medicinal chemistry, and machine-learning technologies to discover and optimize novel compounds for the treatment of cancer^[55]^. COTI-2 was revealed to act, at least in part, *via* the reactivation of mutant p53 to a WT form, involving zinc chelation, in addition to modulation of the PI3K/AKT/mTOR pathway^[56]^. However, COTI-2 has also been reported to act independently of p53, namely by inhibition of the mTOR pathway and activation of AMPK^[57]^. This compound was found to be safe and well tolerated in animal models^[58]^, and it is now being evaluated in a phase Ⅰ clinical trial (NCT02433626) for the treatment of recurring gynecological and other malignancies. Several small molecule classes have been developed to specifically bind to the Y220C pocket. These classes have different chemical structures and have been subjected to different screening methods. Typically, computational platforms, such as chemical structure modeling and molecular docking, are followed by affinity evaluation using biophysical methods, such as isothermal titration calorimetry and surface plasmon resonance.

Formerly, a number of 2-sulfonylpyrimidines, such as PK11000-PK11029 were identified as the arylating agents of surface cysteines of both WT-p53 and p53-Y220C^[59]^. Thus, the lead molecule PK11007 was shown to stabilize the p53-Y220C core domain by up to 3 ℃ in experiments using differential scanning fluorimetry and to induce concentration-dependent upregulation of p53 target genes (*P21* and *PUMA*) in cancer cell lines.

Another group of molecules identified by the same research group are carbazole derivatives that display firstly micromolar affinity *K*_d_ = 150 μmol/L for compound PK-083, 1-(9-ethyl-9H-carbazole-3-yl)-*N*-methylmethanamine^[60–61]^. The carbazole analogue PK-9328 was created through further optimization and its 4-methylthiazole ring was designed to fit into the pocket created by Pro152 and Pro153. This enabled PK-9328 to increase the *K*_d_ by > 70-fold, from 150 μmol/L in PK-083 to 1.7 μmol/L in PK-9328. The co-crystal structure of p53-Y220C with the compound confirmed the additional interactions in the Pro152 and Pro153 pockets^[62]^.

**Figure 3:**
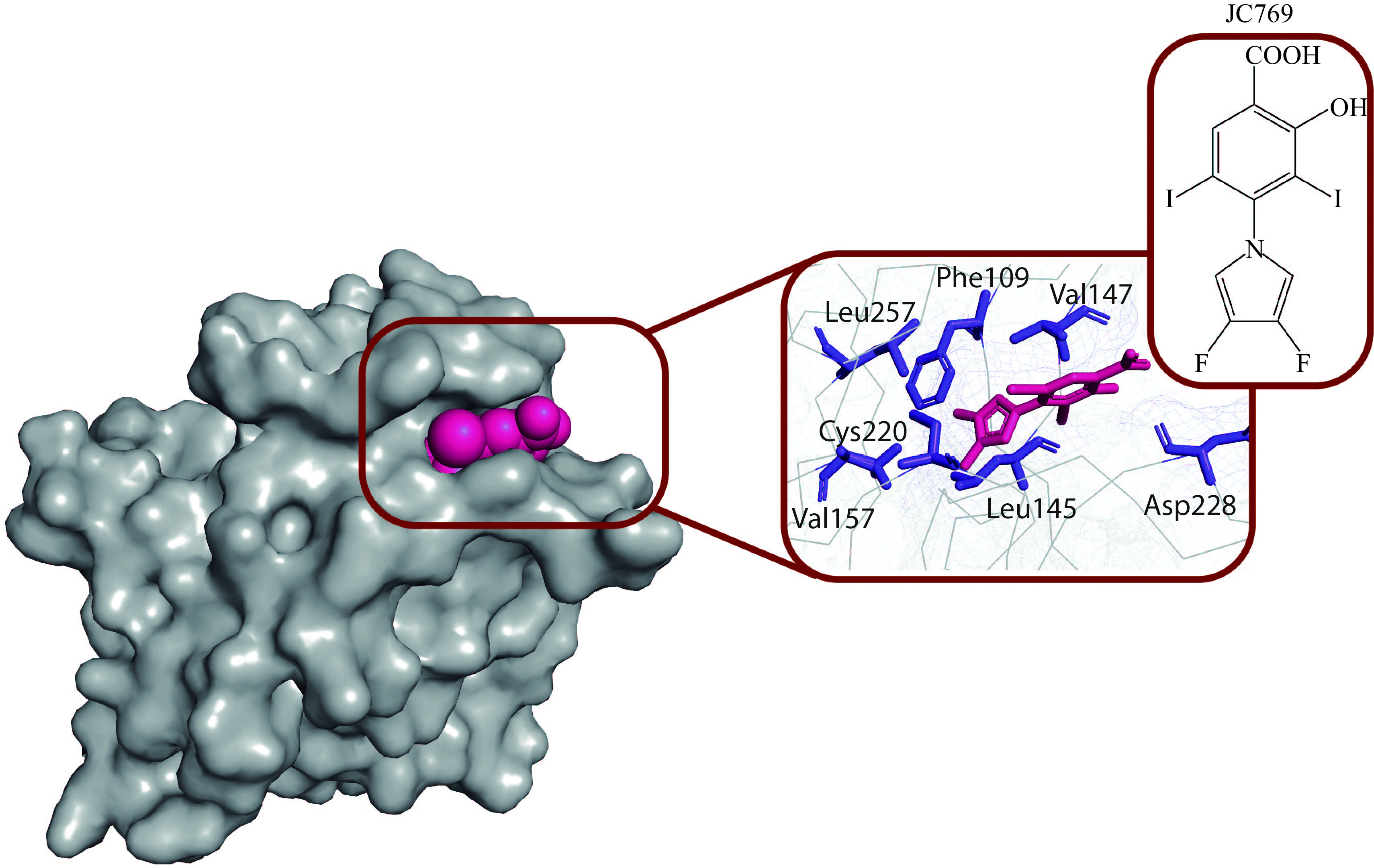
Crystal structure of the p53-Y220C-JC769 complex (PDB code 8A32).

The chemical probe MB710, an aminobenzothiazole derivative, and its ethylamide analog MB725, are the members of the next class of small-molecule stabilizers^[63]^. MB725 was found to exhibit a strong and selective reduction in viability in the p53-Y220C cancer cell lines BXPC-3, HUH-7, and NUGC3 at concentrations below 40 µmol/L, while it maintained a relatively low toxicity in the same concentration range in the p53-R273C mutant cell line SW1088 and the p53 WT cell lines WI38 (normal fibroblast cell line) and NUGC4^[63]^. The reduction of cell viability has been correlated with an increased and selective transcription of p53 target genes, such as *BTG2*, *P21*, *PUMA*, *FAS*, *TNF*, and *TNFRSF10B*, that promote apoptosis and cell cycle arrest, suggesting a compound-mediated transcriptional activation of the Y220C mutant^[63]^. Biophysical and structural characterizations of new MB710/725 analogs have also been recently reported^[64]^, in which a differential scanning fluorimetry was used to evaluate how well these compounds stabilized p53-Y220C, and isothermal titration calorimetry was used to calculate the affinities (*K*_d_ values) of some of the molecules with the highest thermal shifts (Δ*Tm*). Fluorinated compound JC744 was demonstrated *K*_d_ = 320 nmol/L and effectively stabilized the protein, with a difluorinated analog exhibiting similar characteristics^[64]^. Both molecules bind to the Y220C pocket with sub-micromolar *in vitro* affinity, marking a novel promising class of potent and non-toxic compounds for reactivation of the p53-Y220C mutant in an anticancer therapy. The crystal structure of p53-Y220C with an iodophenol-based stabilizer JC769, a member of the same class of molecules, is available (***Fig. 3***).

Derivatives of halogenated phenols also bind to the p53-Y220C pocket^[60]^. Representatives of this class of compounds are the 2-(aminomethyl)-4-ethynyl-6-iodophenols, as exemplified by PK-5196^[65]^, and the substituted 4-phenyl-3-(1H-pyrrol-1-yl)-1H-pyrazole class, as exemplified by the soluble analog PK-7242^[61]^. Halogen-bonding interactions with amino acids inside the cavity are hypothesized to play an important role in the binding. The halogenated compounds slow the rate of thermally induced protein unfolding/aggregation and increase the melting temperature of p53-Y220C^[65]^. The iodinated-phenol core may therefore be a starting point for developing new molecular designs in this area. A series of multifunctional compounds were also designed, which contained the iodinated phenols serving as structural stabilizers of the p53-Y220C surface cavity and Zn-binding fragments for metallochaperone activity^[66]^. The same team also announced the discovery of two novel bifunctional ligands, L5-P and L5-O, designed to act as Zn metallochaperones and non-covalent binders in the Y220C mutant pocket. In both the NUGC3 and NCI-60 Y220C mutant cell lines, the novel ligands were found to be more cytotoxic than the previous compound^[67]^.

There is some evidence regarding the use of natural compounds to rescue the mutant p53-Y220C. Withaferin A and withanone, two withanolides extracted from Ashwagandha (*Withania somnifera*) leaves, were reported to restore WT p53 functions in mutant p53-Y220C cells (HuH-6 and HuH-7), and the induction of p21-WAF-1 associated with apoptosis was observed in both HuH-6 and HuH-7 cells^[68]^. These findings suggest the therapeutic potential of withanolides for the p53-Y220C-harboring cancer cells. Curcumin was also shown to stabilize the mutant p53-Y220C and restore its WT transcription functions by initiating downstream intrinsic and extrinsic apoptotic signaling pathways in the BxPC-3 pancreatic cancer cell line^[69]^. Additionally, caffeic acid phenethyl ester, a bioactive compound from propolis, was found to have the potential to restore WT functions of the mutant p53-Y220C by using bioinformatics computational methods and free energy calculations, similar to a previously described compound PK7242^[70]^. Cell-based experimental data further support the possibility of using this molecule as a potential natural drug for the treatment of mutant p53-Y220C harboring cancers^[70]^. Another bioinformatics study revealed the therapeutic potential of small phytochemicals from *Foeniculum vulgare* (fennel), such as juglalin, retinol, and 3-nitrofluoranthene, to reactivate mutant p53-Y220C^[71]^. Future studies are required to validate their efficacy *in vitro* and *in vivo*.

PC14586, the first orally bioavailable small molecule and selective reactivator of p53-Y220C, is currently undergoing a phase Ⅰ/Ⅱ clinical trial (NCT04585750). This trial investigates the safety, tolerability, and efficacy of multiple dose levels of PC14586 alone and in combination with the immune checkpoint inhibitor pembrolizumab, a monoclonal antibody (mAb) that blocks the interaction between programmed cell death 1 (PD-1) and its ligands PD-L1 and PD-L2. A synergistic antitumor effect was found, when the Y220C activator was combined with anti-PD-1 checkpoint therapy^[72]^. PC14586 with a *K*_d_ of approximately 2.5 nmol/L was shown to stabilize the mutant Y220C, resulting in the reactivation of its transcriptional activity and subsequent expression of its target genes (*e.g.*, *P21*, *MDM2*, *BAX*, and *PUMA*)^[73]^. The high activity of PC14586 was confirmed using cell models and tumor xenograft mice^[51,73]^. PC14586 also showed a preliminary efficacy in patients with advanced solid tumors harboring the p53-Y220C mutation^[74]^.

Finally, KG13, an azaindole-based molecule, was shown to specifically label p53-Y220C but not p53 WT cysteine residues^[37]^. KG13 is also able to activate p53 target genes without affecting DNA-binding specificity^[37]^. Notably, this represents the first allele-specific compound selectively reacting with the cysteine residues of p53-Y220C, restoring WT thermal stability to a level with a Δ*Tm* of + 8.3 ℃(± 0.1 ℃)^[37]^. In line with the needs of precision medicine, the discovery of this compound may improve approaches to generate the p53 WT activity specifically in tumor cells harboring the p53-Y220C mutation.

## Immunotherapy

Recently, the p53-Y220C peptide-human leukocyte antigen complex has been found to be present on the surface of cancer cells, making it a promising target for the targeted cancer immunotherapies^[19]^. Initially, several attempts to generate antibodies against WT and mutant p53 have been made over the past few decades^[75]^. Most of those antibodies interact with epitopes located at the N-terminal and C-terminal regions of the p53 protein^[76]^. However, the centrally located core region is poorly antigenic, and only a few (mAbs) have been generated to be specific to this critical DNA binding. Next, three mAbs have been generated against the p53 DNA-binding core domain^[77]^. Immunoblotting techniques allow all of them to be tested for recognition of the mutant p53-Y220C protein. These data demonstrate the possibility of reproducible generation of amino-acid-specific mAbs against p53-Y220C and their use as therapeutic agents.

Bispecific antibodies represent a class of mAbs capable of simultaneously binding to two antigens. A subtype of bispecific antibodies called bispecific T cell engagers (BiTEs) has been developed to simultaneously bind to tumor-expressed antigens (*e.g.*, BCMA and CD19) and CD3 on T cells^[78]^. BiTEs bind to the target protein on tumor cells and trigger the signaling mediated by the CD3 co-receptor on T cells, thereby activating cytotoxic T cells for subsequent elimination of the tumor cells. BiTEs that have specificity for the peptide-major histocompatibility complex class Ⅰ (pMHCⅠ) of the target cells and CD3 of T or natural killer cells represent a novel class of antibodies, with highly promising therapeutic modalities against cancers associated with the mutant p53^[68]^, and these BiTEs are often referred to as T cell receptor (TCR)-mimic (TCRm) or TCR-like antibodies. TCRm Abs specific to pMHCⅠ presenting WT and mutant p53 antigens have demonstrated encouraging antitumor effects both *in vitro* and *in vivo* animal models^[68–79]^.

**Figure 4:**
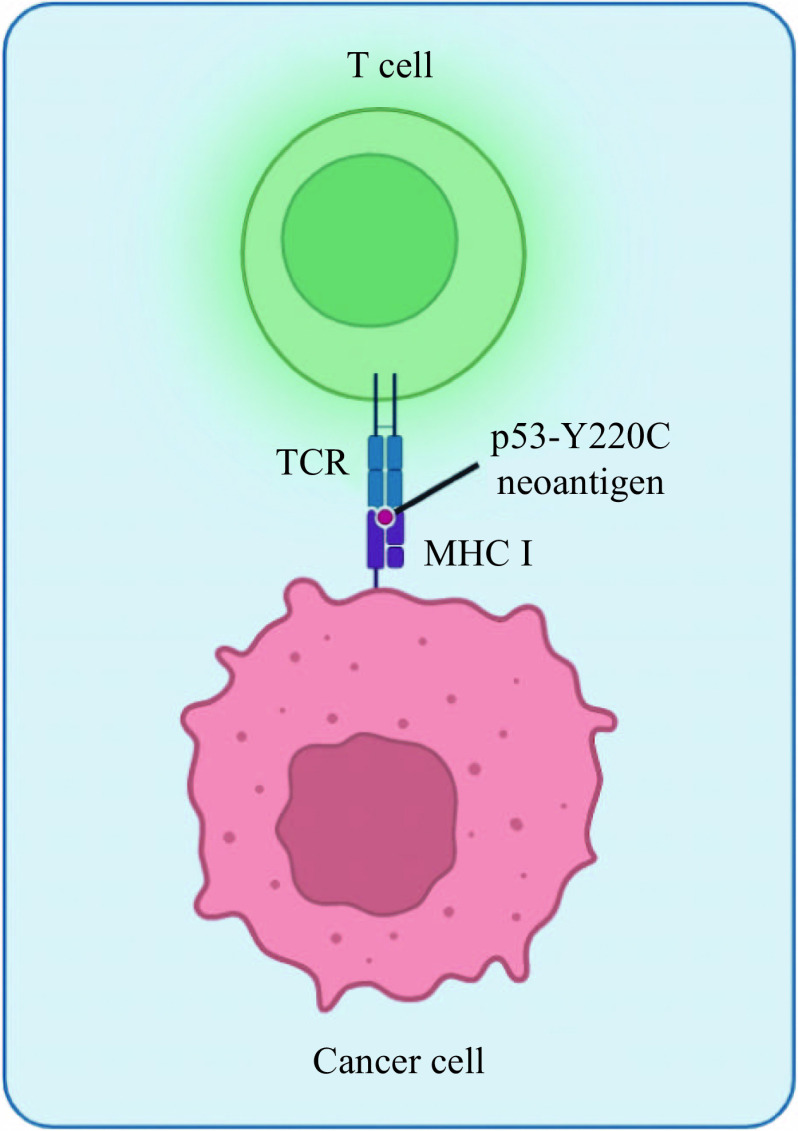
A possible immunotherapy approach to target the tumor cells expressing the p53-Y220C neoantigen.

Bispecific TCRm Abs that recognized tumor cells expressing the p53-R175H neoantigen were reported^[80]^. In the mouse model of multiple myeloma, these BiTEs effectively stimulated T cells to eliminate tumor cells presenting mutant p53 without affecting normal cells with the WT p53. Even when the mutant p53 neoantigens were presented on the surface of the tumor cells at "extremely low" levels, the BiTEs were still able to activate a specific T cell-mediated antitumor response^[80]^. Thus, the utilization of TCRm Abs may be potentially useful to target cancers with other somatic p53 mutations, including Y220C^[81]^. Taking into account the availability of the amino acid sequence of TCRs with antigenic specificity not only for p53-R175H but also for p53-Y220C, it is now possible to develop TCRm Abs recognizing p53-Y220C^[82]^ (***Fig. 4***). In addition, the shared p53-Y220C neoantigen expressed by cancer cells with the amino acid sequence VVPCEPPEV, which may potentially be used to develop such immunotherapeutics, was found to have a low stability and a low affinity of binding to HLA-A0201 molecules; the altered neoantigen, in which VVPCEPPEV was substituted by VLPCEPPEV, was shown to increase affinity and stability and improve immunogenicity^[83]^. In many cases, p53 mutations are associated with significant overexpression of immune checkpoint proteins, such as PD-1, which suggests that these types of tumors may be amenable to anti-PD-1/PD-L1 immunotherapy in addition to other approaches^[84–85]^. Potential approaches include a combination of immunotherapy with immune checkpoint inhibitors, such as antibodies^[86]^. Thus, the employment of TCRm Abs recognizing p53-Y220C may be potentially useful to target solid tumors that lack T cell infiltration, especially in combination with other approaches, such as oncolytic viruses and cancer vaccines that are being evaluated to enhance T cell trafficking to tumors^[87–88]^. There is a reasonable expectation that this ambitious goal will be achieved in the near future.

## Conclusions and perspectives

Although the p53 protein has been long considered undruggable as a therapeutic target, strategies aiming to target p53 have now advanced considerably, generally involving the activation of WT-p53 and the restoration of WT-like functions in the mutant p53. Several compounds have been produced to activate WT-p53 *via* the disruption of its main negative regulators, MDM2/MDMX, which are now being clinically studied and have shown some success in recent years. However, these molecules only work in half of all malignancies, and in the other half, p53 is damaged by mutations and cannot be effectively targeted by treatment with MDM2 inhibitors. A growing number of small molecules have been developed in recent years to promote proper folding and/or reactivation of common missense-mutant p53 proteins. Several of the compounds target missense mutant p53 by binding covalently to cysteine residues in the p53 core domain, improving the protein thermostability. Other compounds have been shown to bind non-covalently and act as Zn^2+^ chelators or prevent the mutant p53 aggregation. DBD of the oncogenic p53 mutant Y220C, destabilized by the mutation, has an extended surface crevice that accommodates therapeutic agents to restore WT-like functions of the mutant p53. The variety of small molecules targeting mutant p53-Y220C previously discovered is encouraging. There is a realistic expectation that at least some of these compounds will prove to be effective in clinical practice. It is also worth noting that the p53-Y220C binder PC14586 has recently entered clinical trials. The progress made in targeting this mutation is based on the information about its structure that provides a proof of concept and a tremendous opportunity to develop new, more effective classes of anti-cancer drugs for this mutation in the future. In addition, there are cases where immunotherapeutic agents, such as mAbs, immune checkpoint inhibitors, and small molecules that reactivate p53-Y220C, are used in combination.

Future studies are needed to determine the effect of combination therapy with therapeutic hypothermia, cell cycle inhibitors, or proapoptotic drugs. Expectations are also pinned on immunotherapeutics, including those expected in the near future. Since *TP53* mutations are found in about half of all human tumors, a precision medicine approach to induce WT p53 activity in tumor cells may be used to treat a large number of human cancers. If such an approach is successfully translated into clinical practice, the potential public health benefits may be substantial.
